# Developing water, energy, and food sustainability performance indicators for agricultural systems

**DOI:** 10.1038/s41598-021-02147-9

**Published:** 2021-11-24

**Authors:** Soheila Zarei, Omid Bozorg-Haddad, Vijay P. Singh, Hugo A. Loáiciga

**Affiliations:** 1 Department of Irrigation & Reclamation Engineering, Faculty of Agricultural Engineering & Technology, College of Agriculture & Natural Resources, University of Tehran, Karaj, Tehran Iran; 2 Caroline & William N. Lehrer Distinguished Chair in Water Engineering, Department of Biological and Agricultural Engineering & Zachry Department of Civil & Environmental Engineering, Texas A&M University, 321 Scoates Hall, 2117 TAMU, College Station, TX 77843-2117 USA; 3 Department of Geography, University of California, Santa Barbara, CA 93106 USA

**Keywords:** Biological techniques, Ecology, Climate sciences, Ecology, Environmental sciences, Environmental social sciences, Hydrology, Natural hazards, Engineering, Mathematics and computing

## Abstract

Water use by the agricultural sector along with inefficient irrigation methods and climate change has led to the depletion and insecurity of water resources and consequent instability of the agricultural system. Defining benchmarks and comparing them is essential for sustainable system management performance. The sustainability performance of an agricultural system depends on various factors related to water, energy, and food. This study selects and ranks sustainability performance indicators (SPIs) of agricultural systems with the analytical hierarchy process (AHP). Expert opinions on agricultural sustainability were obtained from Iran’s Regional Water Organization. The factors and variables affecting the management of water resources in agricultural systems in a basin area are evaluated with 17 SPIs (10 indicators of water resources sustainability, 3 energy sustainability indicators, and 4 food sustainability indicators) that measure the sustainability of agricultural systems. The AHP reduced the number of indicators to a small number of effective indicators. Results of pairwise comparison and the subsequent determination of the weight of each indicator show that the indicators of water consumption, groundwater level stability, vulnerability of water resources, and water stress have the largest weights (i.e., importance) for agricultural system sustainability at the basin scale. These selected indicators can be applied to agricultural water systems (AWSs).

## Introduction

The availability of water resources has been on the decline in recent decades, with the reduction in steady river flow and the depletion of groundwater to meet the needs of growing populations. Water scarcity coupled with inefficiency has impacted the agricultural sector, which is a major consumer of water resources, and has consequently put food security at risk. Water scarcity and food security are among the factors that cause environmental instability^1^. Sustainable agricultural development protects land, water, animal and plant resources in an environmentally friendly manner without degradation that is technically appropriate, economically reasonable, and socially acceptable (Food and Agriculture Organization of the United Nations^2^).

Sustainable agricultural and rural development revolve around three main pillars: food security, job creation, and income generation to eradicate poverty and preserve natural resources and the environment^3^. Food security is a function of economic, social, natural, political, and cultural factors which are affected by agricultural production policy, food distribution system, natural resources, consumption and nutrition pattern, employment and income distribution status, foreign trade policy, and nutrition culture. Therefore, agriculture impacts various economic, social, political, environmental and other dimensions. While properly managing and using resources to meet food needs, sustainable agriculture increases the quality of environment and reserves of natural resources. Sustainable development in rural areas is complicated by its interaction with economic, social, environmental, technical, and political goals^4^.

Sustainable agricultural development experiences the challenges of severe erosion of soil resources, waste and inefficient use of surface and groundwater resources, degradation of vegetation, and unprincipled use of natural resources, high waste of agricultural products (in production, transport, storage, distribution, and consumption stages), unemployment in rural areas, dichotomy in the structure of urban and rural areas, rural development management, and achieving investment security in the productive sectors. Thus, giving the importance of sustainable agricultural development there is urgency in assessing agricultural sustainability^5^. Water resources management is a basic tool to achieve food security, prevent uncontrolled soil erosion, and prevent water loss in the agricultural sector. World Economic Forum^6^ explains that water systems bear some similarities with food, climate and economic systems, and that these similarities must be exploited in order to maximize benefits. It is therefore imperative to evaluate the structure of the Nexus between water systems and other systems with integrated sustainability indicators for effective policy making.

Bassel et al.^7^ developed the WEF Nexus modeling tool (WEF Nexus Tool 2.0) to evaluate different scenarios and to study sustainable resource allocation strategies. It is a comprehensive framework that reflects the multidimensional, interdisciplinary nature of resource management projects. This study was conducted for the study area of Qatar. Quantitative tools are now available for environmental impact assessment and cost–benefit analysis^8^. Sustainability of the agricultural sector can be assessed using sustainability performance indicators (SPI) which then can be used in the management of agricultural systems. The UN Committee on Sustainable Development has categorized the indicators of sustainable development into social, economic and environmental groups. Due to the increase in stakeholders, a comprehensive tool for analyzing various aspects is therefore needed^9^. Since water supply decision-making involves several criteria, models, and data sources^10^, multi-criteria decision making can be applied to manage water resources^11^, in the areas of watershed management^12,13^, drainage control^14,15^, wastewater treatment and disposal^16,17^, and water supply^18^.

Effective indicators used for the management of agricultural water resources in different regions can be useful in the development planning of those regions. Developing appropriate sustainability indicators helps policymakers identify weaknesses in various economic, social, and environmental aspects of future planning and for sustainable and comprehensive development. The use multi-criteria decision making may assist in achieving water sources sustainability^19^. This study therefore applies the analytical hierarchy process (AHP) for multi-criteria decision making.

AHP is based on pairwise comparisons of alternatives and has been used in water resources^20^ implemented this method to evaluate non-conventional water resources supply alternatives to ensure sustainability in Jordan. Using AHP^21^ concluded that groundwater recharge was the best alternative for wastewater reuse. Ilaya-Ayza et al.^22^ evaluated water supply considering the criteria of water pressure, number of users, number of supply hours, and ease of operation in AHP.

This study investigates the integrated assessment of agricultural sustainability from the perspective of three dimensions of water, energy, and food and their environmental impacts. Sustainable development depends on the sustainability of water resources and the link between society and environment. This study relies on water, food, and energy SPIs to assess the sustainability of the regional agricultural sustainability. The integrated assessment and decision-making based on the water-energy-food nexus is imperative to support resources management due to the wide range of economic, social, and environmental stakeholders. This work evaluates (i) the sustainability of the agricultural system with a set of SPIs in the water, energy, and food sectors, and (ii) the impact of each sector on the sustainability of the other sectors by the AHP multi-criteria decision-making method based on water-food-energy nexus.

## Methodology

### Case study

**Figure 1:**
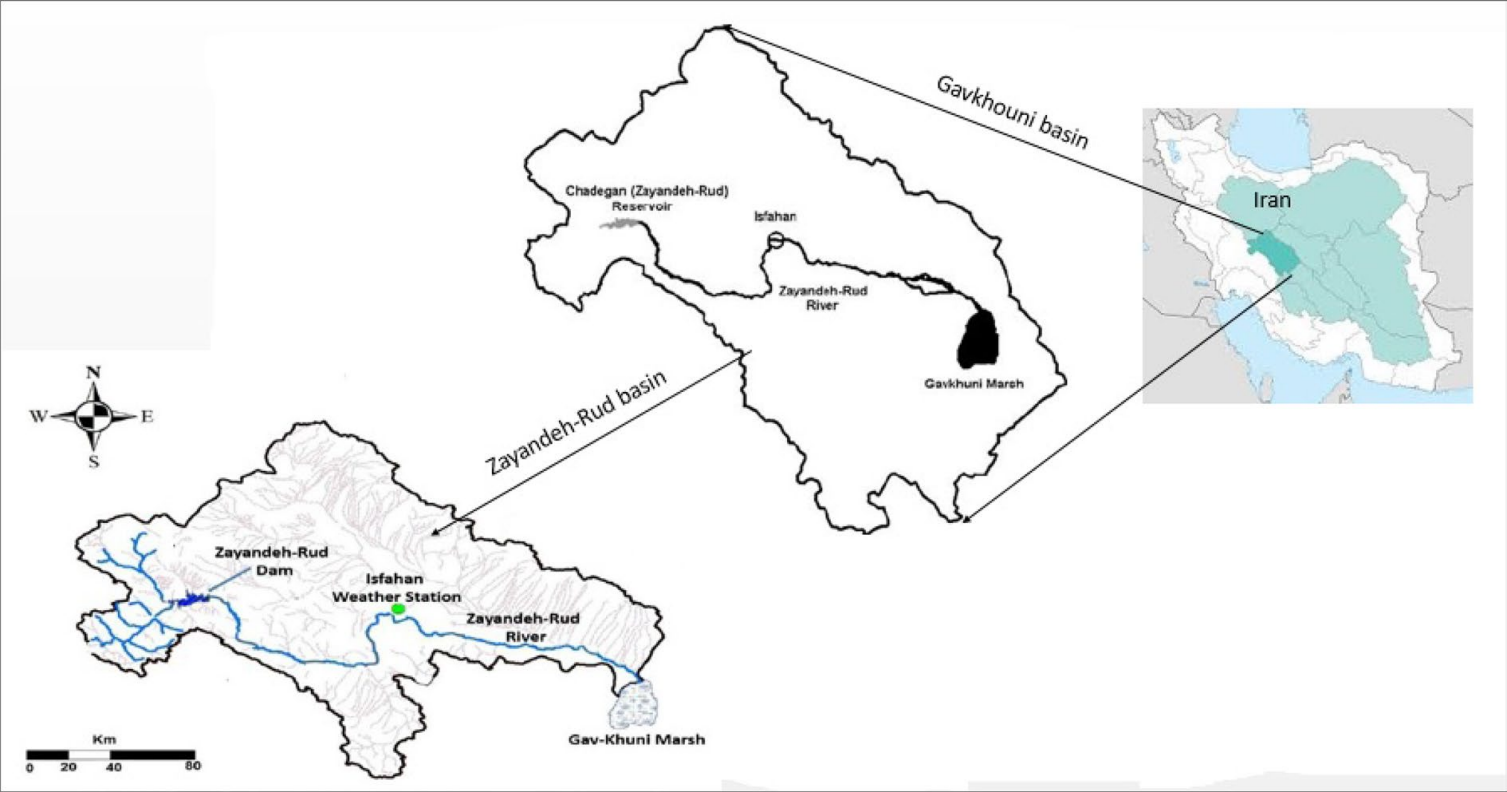
The location of the Zayandeh-Rud basin in Iran.

The Zayandeh-Rud basin (Fig. 1), a arid region of Iran, was selected to evaluate the SPIs. The Zayandeh-Rud basin is located in the central part of Iran. It has an area of 26,972 km^2^ area, where there are multiple water stakeholders such as agriculture, industry, urban and the environment sectors, with agriculture being the main user of the basin. Water resources in the basin are divided into surface water and groundwater. Approximately 100,000 ha among 113,000 ha of the agricultural area is irrigated by Zayandeh-Rud dam, and 3100 mm^3^ of water resources are used in the agricultural sector. The main surface water source in the basin, Zayandeh-Rud River originates in the Zagros Mountains and is about 350 km long in a west to east direction passing by the city of Isfahan. The Zayandeh-Rud River is an important water source for the agricultural, industrial, health, and urban sectors in Central Iran and the Chaharmahal-Bakhtiari and Isfahan provinces.

### Multi-criteria decision making

Multi-criteria decision making includes two categories of multi-objective decision making and multi-criteria decision making, which are implemented to select the best decision among several alternatives or to evaluate decisions. This work applies decision making as a multi-criteria decision to achieve a goal. Each decision includes objectives, alternatives, and criteria. A problem’s goal is first defined. Alternatives are different options for wastewater management in this instance that are assigned weights based on their contribution to achieving the goal. Criteria are also factors that are measured by the purpose of the alternatives^23^. The AHP method helps achieve a defined goal after completing the steps outlined below.

### The AHP method

The Analytical Hierarchy Process (AHP), developed by Saaty^24^, is a multi-criteria decision-making method for solving complex problems. It combines objective and quantitative evaluation in an integrated manner based on multi-level comparisons, and helps organize the essential aspects of a problem into a hierarchical format. It regularly organizes tangible and intangible factors and offers a structured and a relatively simple solution to decision problems. The AHP method ranks alternatives propose to tackle a decision-making problem. The ranking is based through a sequence of pairwise comparisons of evaluation criteria and sub-criteria.

#### The AHP structure

**Figure 2:**
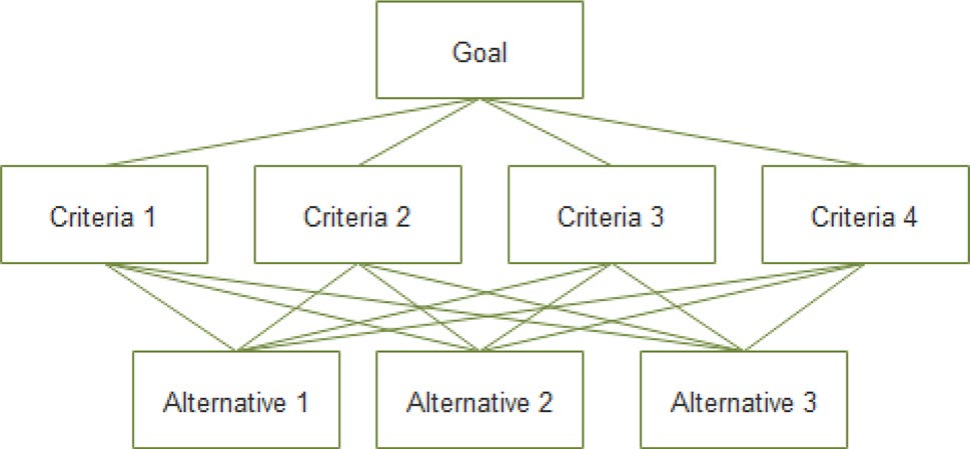
Goal, criteria, and alternatives in a generic hierarchical structure.

In a hierarchical structure the communication flow is top-down. First, indicators and evaluation criteria are defined from experts who are asked for their expert opinions. The criteria serve the purpose of determining the relative worth of alternatives entertained to solve a multi-criteria decision-making problem. Thereafter, the problem is divided into criteria and sub-criteria for the evaluation of alternatives. Figure 2 depicts a generic AHP structure depicting a goal to be met with $n$ = 4 evaluation criteria, and $m=3$ alternatives to cope with a problem (in our case SIPs).

#### The pairwise comparison matrix

The pairwise comparison matrix ($$A$$), called the Saaty Hierarchy Matrix, measures the importance of each criterion (or sub-criterion) relative to other criteria based on a numeric scale ranging from 1 to 9. Criteria that are extremely preferred, very strongly preferred, strongly preferred, moderately preferred, and equally preferred are assigned the values 9, 7, 5, 3, and 1, respectively, in the scale of preference; intermediate values are assigned to adjacent scales of preference. Thus, the values 8, 6, 4, and 2 are assigned respectively to the adjacent scales (9,7), (7,5), (5,3), and (3,1)^24^. These numerical assignment of values is made based on the opinion of experts^25^. The pairwise comparison matrix ($$A)$$, therefore, represents a set of relative weights assigned to the criteria^23^. The general form of a pairwise comparison matrix when there are $$n$$ evaluation criteria is written in Eq. (1):

1 $$A=\left[{a}_{ij}\right]=\left[\begin{array}{cccc}{1=w}_{1}/{w}_{1}& {w}_{1}/{w}_{2}& \dots & {w}_{1}/{w}_{n}\\ {w}_{2}/{w}_{1}& 1={w}_{2}/{w}_{2}& \dots & {w}_{2}/{w}_{n}\\ .& .& \dots & .\\ .& .& \dots & .\\ .& .& \dots & .\\ {w}_{n}/{w}_{1}& {w}_{n}/{w}_{2}& ...& 1={w}_{n}/{w}_{n}\end{array}\right]$$

where ${w}_{i}/{w}_{j}$ denotes the weight assigned to the $i$-th criterion relative to the $j$-th criterion^24^. Clearly, ${a}_{ji}=1/{a}_{ij}$, with ${a}_{ji}={a}_{ij}=1$ when $i=j$.

#### The ratio matrix

The ratio matrix ($R$) has elements ${r}_{ij}$ is calculated by Eq. (2):

2 $$R=\left[{r}_{ij}\right]=\left[\begin{array}{cccc}1& {a}_{12}& \dots & {a}_{1n}\\ 1/{a}_{12}& 1& \dots & {a}_{2n}\\ .& .& .& .\\ .& .& .& .\\ .& .& .& .\\ 1/{a}_{1n}& 1/{a}_{2n}& \dots & 1\end{array}\right]$$

clearly, ${r}_{ij}={a}_{ij}$ when $j\ge i$, and ${r}_{ij}=1/{a}_{ji}$ when $j<i$. The 4 × 4 ratio matrix obtained for the criteria of relevance, measurability, data availability, and comparability is given by Eq. (3):

3 $$R=\left[\begin{array}{cccc}1& 2& 2& 2\\ 1/2& 1& 1& 3\\ 1/2& 1& 1& 2\\ 1/2& 1/3& 1/2& 1\end{array}\right]$$

The ratio matrix is instrumental in calculating the criteria weights.

#### Determining the criteria weights

The weights assigned to the criteria must be determined. For this purpose the ratio matrix is multiplied by the vector of weights (${\varvec{w}}$) as shown in Eq. (4):

4 $$R {\varvec{w}}= {\lambda }_{i} w or \left[\begin{array}{cccc}1& {a}_{12}& \dots & {a}_{1n}\\ 1/{a}_{12}& 1& \dots & {a}_{2n}\\ .& .& .& .\\ .& .& .& .\\ .& .& .& .\\ 1/{a}_{1n}& 1/{a}_{2n}& \dots & 1\end{array}\right]\left[\begin{array}{c}{w}_{1}\\ {w}_{2}\\ .\\ .\\ .\\ {w}_{n}\end{array}\right]={\lambda }_{i}\left[\begin{array}{c}{w}_{1}\\ {w}_{2}\\ .\\ .\\ .\\ {w}_{n}\end{array}\right]$$

where ${\lambda }_{i}$ is denotes a component of the vector of eigenvalues $\lambda$. The system of Eqs. (4) represents the classic eigenvalue problem that is solved for $\lambda$ from the equation $\left|R-\lambda I\right|$= 0 were $I$ represents the $n \bullet n$ identify matrix and | | denotes the determinant of a matrix. For each element ${\lambda }_{i}$ of $\lambda$ there is a corresponding vector ${{\varvec{w}}}_{{\varvec{i}}}$, $i=1, 2, \dots ., n.$ The largest eigenvector is denoted by ${\lambda }_{max}$ and its corresponding vector ${\varvec{w}}$ contains the weights assigned to the criteria. The weights so developed are normalized to add to one as discussed in the Results and Discussion section. Weighting vectors were also calculated for 17 sustainability performance indicators (SPIs) with respect to the evaluation criteria. The procedure to calculate the weights for each SPI is the same as described in this section. See the calculated weights in the Results and Discussion section.

#### The consistency ratio

The consistency ratio ($CR$) is calculated to determine the acceptability of the weights determined according to the previous section. First calculate the consistency index ($CI$) as Eq. (5):

5 $$CI= \frac{{\lambda }_{max}-n}{n-1}$$

According to Eq. (6), the consistency rate ($$CR$$) is also obtained by dividing the consistency index by the random index ($$RI)$$:

6 $$CR= \frac{CI}{RI}$$

The consistency rate is an indicator that shows possible inconsistencies in the pairwise comparison matrix. It takes the value 0 (complete consistency) when ${\lambda }_{max}=n$. The random index takes the values 0, 0, 0.58, 0.9, 1.12, 1.24, 1.32, 1.41, 1.45, 1.49 corresponding to the number of criteria *n* = 1, 2, 3, 4, 5, 6, 7, 8, 9, 10, respectively^24^. The acceptable *CR* level should not exceed 10%, but some studies have suggested that the acceptable *CR* levels may be up to 20%^27,28^.

### Selection of the sustainability performance indicators (SPIs)

One of the most important elements of agricultural systems is water resources^29^. Various indicators of the sustainability of agricultural systems were considered. As mentioned earlier, the United Nations (UN) has classified sustainability indicators into three categories: economic, social, and environmental. Sustainable development has been widely used since the 1980s to address the negative consequences of development and policies on the environment and society^30^. Also, the UN has defined 17 Sustainable Development Goals (SDGs) in 2015 to achieve 2030 sustainable development program, which address global challenges such as poverty, environmental degradation, justice, and more. In this research, sustainable indicators involve the water, energy, and food sectors to embed sustainable development concept in the water-energy-food nexus.

Seventeen SPI were selected; 10, 3, and 4 corresponding to the water, energy, and food, sectors, respectively. The selected SPIs are listed in Table 1. Sustainability performance indicators were identified considering multiple attributes of sustainability (sustainable water resources, sustainable agriculture, sustainable development, environment, yield sustainability, food sustainability, and energy sustainability). In fact, the sustainability performance indicators of the Table 1 are connected to the UN SDGs. According to the UN Sustainable Development Goals (https://sdgs.un.org/goals), the SDGs include access to safe water and sanitation for all, food supply and eradication of poverty, job creation and income for young people, social welfare, the provision of affordable and clean energy, and more. The indicators in Table 1 are also a set of assessment criteria for assessing water, food, income, and energy supply, as well as assessing the environment by examining water stress, reliable water supply, available water, groundwater level sustainability, and greenhouse gas emissions. Calculating each of the sustainable indicators is described at the following, and all these indicators are used in different parts of an agricultural system.

**Table 1:** Selected SPIs.

	Index
Water	Water stress
	River flow index in the dry season
	Reliable water supply
	Groundwater level sustainability
	Irrigation performance index
	Water consumption per kg of product
	Available water index
	Water efficiency index
	Water economic efficiency index
	Water resources vulnerability index
Energy	Energy performance index
	Energy sustainability index (ESI)
	GHG emissions from energy use
Food	Food security index
	Revenue index
	Price Index
	Farm Net Value Added (FNVA)

#### Water stress

The water stress index ${HWSI}_{i,t}$ is given by Eq. (7), which quantitatively evaluates the stress on water resources:

7 $${HWSI}_{i,t}= \frac{{Supply}_{i,t}}{{Population}_{i,t}}$$

where, $i$: consumer number, $t$: time of consumption, Supply: the amount of meeting the needs of the consumer $i$ at time $t$ (MCM), population: consumer $i$ population at time $t$.

#### River flow index in the dry season

This index was developed by the World Resources Institute^31^ to describe water conditions in a river basin. This index calculates the timing of changes in water access in different seasons, such as the dry and wet seasons. Basins in a dry season have less than 2% of the surface runoff in four months of the driest months of the year (the sum of the lowest runoff during four consecutive months). This index is expressed by Eq. (8):

8 $$River\,flow\, index \,in \,the \,dry \,season = \frac{Runoff \,volume\, in\, the\, dry \,season }{Population}$$

#### Reliable water supply

Reliable access to safe drinking water is essential for social and economic sustainable development. The water supply reliability is defined as the ratio of the amount actually supplied to what is provided in the absence of failure (the demand)^32^, and is calculated with Eq. (9):

9 $$Rel=\frac{number \,of \,satisfactory\, conditions}{total\, number \,of \,conditions}$$

#### Groundwater level sustainability

The groundwater level sustainability index $SI$, a function of performance indices^33^, is calculated with Eq. (10):

10 $$SI \, = Rel\, \times \, Res \, \times \, \left( {1 - Vul} \right)$$

where $Rel$, $Res$, and $Vul$ denote reliability. resiliency, and vulnerability, respectively, of groundwater supply^33,34^.

#### Irrigation performance index

The irrigation performance index $SGVP$ or "Standardized Gross Value of Production" (SGVP) is calculated with Eq. (11):

11 $$SGVP= \left(\sum Crops {A}_{i}{Y}_{i}\frac{{P}_{i}}{{P}_{b}}\right){P}_{world}$$

where, A_i_ = Area under plant cultivation (i), Y_i_ = Crop yield (i), P_i_ = Local crop (i) price, P_b_ = Local price of the base crop (dominant crop of a region that has an international market), P_world_ = International price of the crop base. The SGVP allows the performance of irrigation schemes to be compared regardless of the location and type of crop planted.

#### Water consumption per kg of product

Water is an essential environmental factor that contributes to sustainable economic growth. Water, being limited resource, is a key input that must be included in the sustainability assessment of water use in agriculture. An effective factor in water efficiency can play a significant role in estimating the level of sustainability. Therefore, the level of water use was estimated through irrigation. The water footprint indicator shows the amount of water consumed per unit of product obtained. The lower the value of this index, the more sustainable production is^35^.

#### Available water index

Temporal changes in available water were calculated by Meigh et al.^36^. This index includes surface water and groundwater resources, and their differences in terms of demand for all urban, industrial and agricultural sectors. This index is calculated with Eq. (12) and its value ranges between 1 and − 1.

12 $$Available \,water\, index = \frac{(S + G-D) }{(S+ G+D)}$$

where $S$= surface water volume, $G$ = groundwater volume, and $D$ = the sum of the water demands of all sectors. An index equal to zero means supply and demand are equal.

#### Water efficiency index (kg m^−3^)

This index is obtained with Eq. (13):

13 $$WPC=\frac{1}{VWC}=\frac{CY}{CWR}=\frac{CY }{{\mathrm{Ir}}_{\mathrm{c}}+{\mathrm{p}}_{\mathrm{ec}}}$$

where $Irc$ , p_ec_, and $CY$ denote respectively the water requirement for plant irrigation, the amount of water that comes from rainfall during the growing season, the crop yield.

#### Water economic efficiency index

Economic efficiency refers to the concept of the value of the product material per cubic meter of water applied; it is calculated b Eq. (14):

14 $$BPD=\frac{{I}_{N}}{CWR}$$

where $BPD$, $CWR$, and $IN$ denote respectively the economic productivity of water (Rials per cubic meter), the gross income obtained from the sale of a crop grown in a season (in Rials), and the amount of water applied to grow the crop.

#### Water resources vulnerability index

Gleick^37^ developed this index for basins in the United States as part of an assessment of the effects of climate change on water resources and systems. This index describes the vulnerability of water resources systems based on five criteria and thresholds, each of which is briefly described below. A number of vulnerable regions are identified in each area. This approach emphasizes parts of the basin that are at risk.

This index is obtained by dividing the excess runoff in 5% of a period of study by the excess runoff in 95% in the period of study. Low levels of this ratio indicate low runoff changes and therefore a low risk of floods or droughts. A value greater than 3 indicates the basin is vulnerable to floods and drought.

#### Energy performance index (EPI)

EPI is the energy used per unit area per year or per per capita per year, and is measured in kWh/m^2^/year or kWh/person/year.

#### Energy sustainability index (ESI)

Brown and Sovacool^8^ developed an energy sustainability index based on 12 indicators including four dimensions of oil security, electricity reliability, energy efficiency, and environmental quality. This index is an attempt to measure the sustainability in environmental and energy systems.

#### GHG emissions from energy use

Greenhouse gas emissions per farm (tonnage equivalent to carbon dioxide, for example, tCO2) are a major target, and using the Level 1 and Level 2 procedures of the Intergovernmental Panel on Climate Change—IPCC, it is estimated that the index desirable value must be low, in which a farm produces efficiently insofar as greenhouse gases emissions is concerned. The emission index provides useful information on applied production methods and, more broadly, on agricultural systems. In addition, it supports long-term greenhouse gas emissions assessments, and it contributes to the Common Agricultural Policy (CAP) to reduce climate change.

#### Food security index

The FSI was introduced by IFAD^38^. In general, this index is used to estimate food security at the national level. The FSI is calculated by Eq. (15):

15 $$FSI=0.77 \times \left[\left\{\frac{{x}_{1}}{1+ {x}_{6}}\right\}{\left(1+ {x}_{2}\right)}^{n}\right]+0.23 \times \left[{x}_{4}\left\{\frac{{x}_{s}}{\left(1+ {x}_{s}\right)}\right\}\right]$$

where, ${x}_{1}$, ${x}_{2}$*,* ${x}_{3}$, ${x}_{4}$, and ${x}_{5}$, and ${x}_{65}$ denote the per capita supply of calories per day relative to the required calories, the annual growth rate of calories per capita per day, the food production index, the self-sufficiency index, changes in production, and changes in food consumption, respectively.

#### Revenue index

The revenue index is calculated with Eq. (16):

16 $$Revenue \,Index= \frac{Income \,from \,agricultural\, consumer \,i\, at\, time \,t \,(currency)}{Expected\, income\, from \,agricultural\, consumer\, i \,at\, time\, t\, (currency)}$$

Revenue index equal to or larger than 1 means income equals or exceeds expectations**.**

#### Price Index

The price index is calculated based on the Laspeyres formula, which means that the prices of selected goods in the current period are compared with their prices in the base period (i.e., average annual price in the base year)^39^.

#### Farm Net Value Added (FNVA)

The farm's net value added index is the reward for the use of fixed factors of production (labor, land, and capital). A profitable farm has a large value of this index^40^.

### Criteria for ranking the SPIs

The criteria of relevance (importance), measurability, data availability, and comparability were applied to ranking the SPIs^41–43^. A description of the criteria is found in Table 2.

**Table 2:** Selected criteria for assessing the SPIs.

Criteria	Scale
**Relevance (importance)**	
Very high	The index is highly relevant and more comprehensive indicator of sustainability of agricultural systems
High	The index is highly relevant and of average comprehensiveness
Medium	The index is of average relevance and average comprehensiveness
Low	The index has low relevance to the sustainability of agricultural systems
Very low	The index seems to be irrelevant for the sustainable development of agricultural systems
**Measurability**	
High	Variables have absolute values or one annual observation provides the data for the variables
Medium	Variables have highly variable values that require a large number of observations in a year
Low	These are qualitative data or have estimated values
**Data availability**	
High	Data are available in public annual municipal reports and official water master plan
Medium	Data are available in raw form in internal official records
Low	Data are only available in occasional study reports or rarely available
**Comparability**	
High	The index has been used for agricultural water sustainability assessment in the region (country)
Medium	The index has been used for agricultural water sustainability assessment outside the region
Low	The index has rarely been used for agricultural water sustainability assessment

(References^39–42^).

## Results and discussion

### AHP development

**Figure 3:**
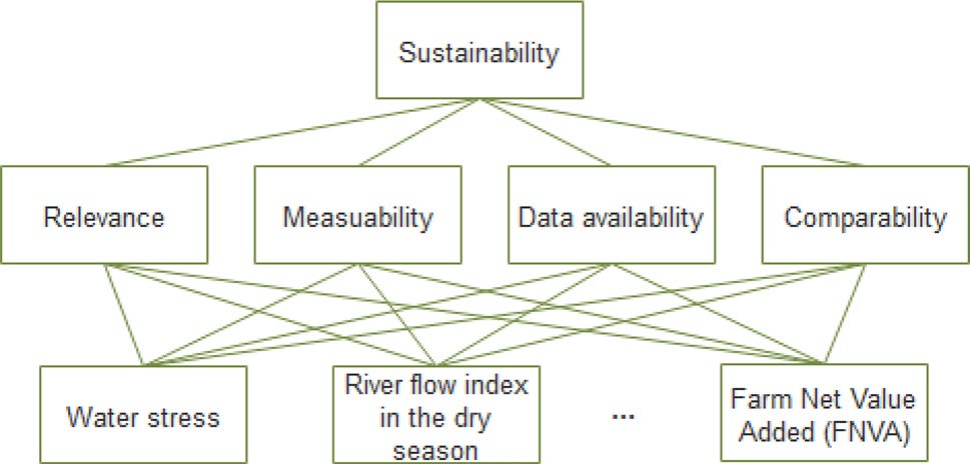
The hierarchy adopted in this study. It has one goal (sustainability, the first level), four criteria (intermediate level) and 17 SPIs (third or inferior level).

SPIs are selected as a combination of indicators of water, energy and food sectors so that the future conditions of agricultural systems can be examined by changing water, energy and food demands, and supply and demand sustainability. Figure 3 presents AHP structure for this study with one goal, four criteria, and 17 SPIs. As can be seen from Fig. 3, sustainability indicators are placed in the last level of the hierarchical structure to be selected through the evaluation criteria of the best indicators to achieve the goal of sustainability in the water, energy, and food sectors. Similarly, according to the United Nations Sustainable Development Goals (SDGs), this structure can be formed in such a way that the first level includes the goal of sustainable development. The goal is related to the last level through the criteria level, which includes indicators for the realization of the 15-year plan for sustainable development in 2030. These indicators, which assess each of the 17 different goals in achieving sustainable development, are prioritized. The selection criteria are weighed for different regions based on their sensitivity and degree of importance base on the opinion of experts.

First, the weights (priority) related to the criteria (level 2 of Fig. 3) must be determined by the AHP method. The weights of the criteria must be determined to clarify the importance of each criterion in achieving the desired goal. For this purpose, a pairwise comparison matrix of criteria, which is a 4 × 4 matrix, was formed and the corresponding special vector was determined according to the weight of the criteria. Normalizing this particular vector determines the weight matrix of each criterion. The normalized weights for the relevance, measurability, data availability, and comparative criteria are listed in Table 3. Clearly the weights add up to 1. Next, the weights of each SPI were determined with respect to the relevance, measurability, data availability, and comparative criteria. The SPIs’ weights are listed in Table 4. The weights (priority) related to the SPIs were calculated in the same way that the weight of the criteria was determined, that is, by forming a matrix A (pairwise comparison matrix) in which each of the SPIs is placed opposite each other and compared, according to the experts’ judgments. These steps are fully described in Sect. 2.2. The sixth (last) column of Table 4 lists the overall weights of each SPI, which were obtained by multiplying the weights of each SPI with respect to the four criteria by the corresponding criteria weights listed in Table 3. Thus, for example, the overall weight for the water-stress SPI = 0.085 × 0.391 + 0.070 × 0.257 + 0.05 × 0.226 + 0.082 × 0.126 = 0.0729. The numbers in this column (the sixth column) indicate the priority of the SPIs in establishing the sustainability of agricultural systems in the three sectors of water, energy and food. The consistency rate (CR) was 0.04, indicating the acceptability of results.

**Table 3:** Normalized weights of the criteria.

Criteria	Weights
Relevance	0.391
Measurability	0.257
Data availability	0.226
Comparative	0.126

**Table 4:** Weights of the SPIs.

	Relevance	Measurability	Data availability	Comparative	Overall weights	Rounded weights
Water stress	0.085	0.07	0.05	0.082	0.072857	0.073
River flow index in the dry season	0.026	0.123	0.103	0.087	0.076017	0.076
Reliable water supply	0.079	0.04	0.075	0.066	0.066435	0.066
Groundwater level sustainability	0.092	0.11	0.049	0.062	0.083128	0.083
Irrigation performance index	0.07	0.088	0.063	0.075	0.073674	0.073
Water consumption per kg of product	0.031	0.143	0.133	0.079	0.088884	0.089
Available water index	0.067	0.063	0.087	0.076	0.071626	0.072
Water efficiency index	0.103	0.053	0.063	0.065	0.076322	0.076
Water economic efficiency index	0.025	0.055	0.047	0.051	0.040958	0.041
Water resources vulnerability index	0.151	0.032	0.047	0.047	0.083809	0.084
Energy performance index	0.028	0.02	0.021	0.043	0.026252	0.027
Energy sustainability index (ESI)	0.034	0.021	0.018	0.025	0.025909	0.026
GHG emissions from energy use	0.087	0.046	0.043	0.027	0.058959	0.059
Food security index	0.054	0.02	0.019	0.021	0.033194	0.034
Revenue index	0.027	0.051	0.081	0.064	0.050034	0.05
Price Index	0.021	0.042	0.06	0.067	0.041007	0.041
Farm Net Value Added (FNVA)	0.02	0.023	0.041	0.063	0.030935	0.031

**Figure 4:**
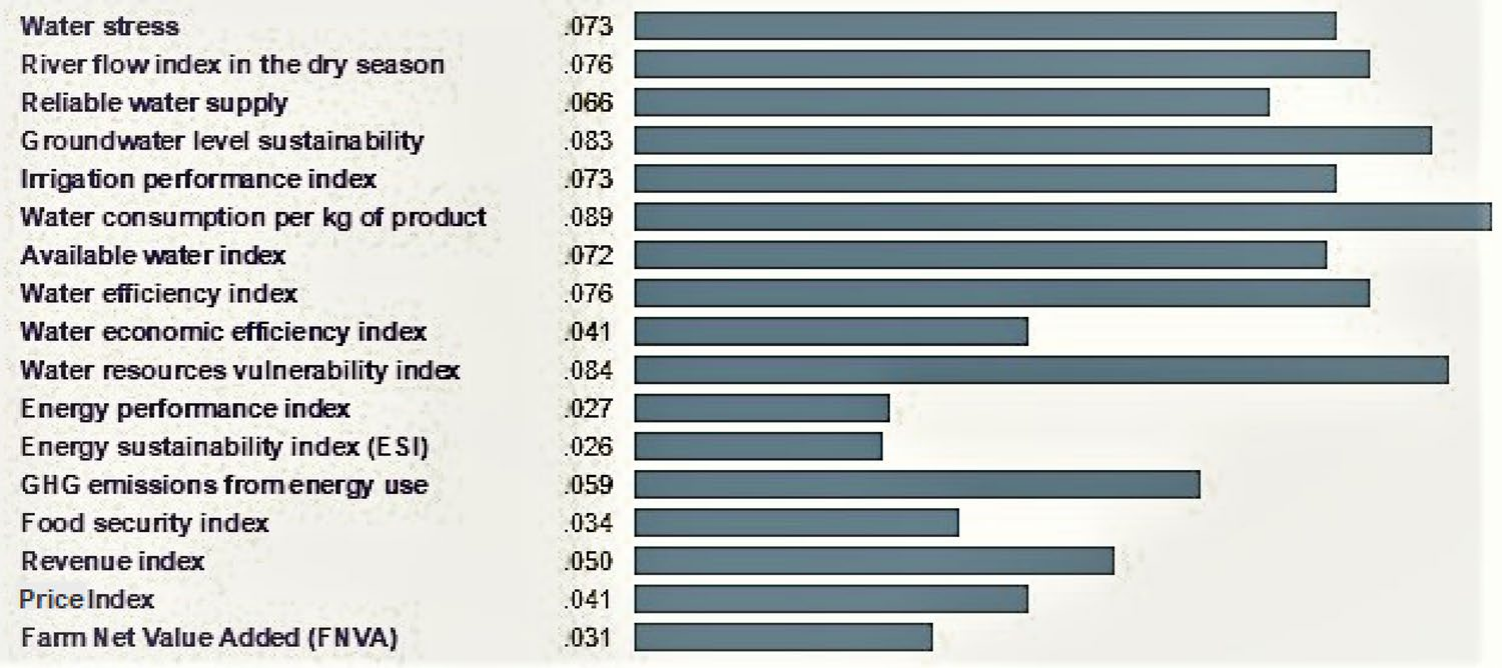
Comparison of alternatives.

Figure 4 shows a graphical comparison of the SPIs in this study. It is seen in Fig. 4 that water consumption per kg of product had the largest weight, and the energy sustainability index (ESI) had the smallest weight affecting the sustainability of the agricultural system. The agricultural sector has the capacity to solve many economic and social problems. Selection of appropriate indicators as a tool to assess the sustainability of agricultural systems and evaluate and compare different executive options is essential. Details of the selected SPIs are described below for each section of water, energy and food.

### The water sector

Providing community health and providing clean and hygienic water are among the UN’ SDGs. The agricultural sector, on the other hand, is a major consumer of water for food supply, which upsets the balance of supply and demand for water resources and creates the problem of water shortage. Therefore, determining the key indicators to assess the appropriate amount of water consumption in the agricultural sector while not harming natural resources and food security is imperative.

**Figure 5:**
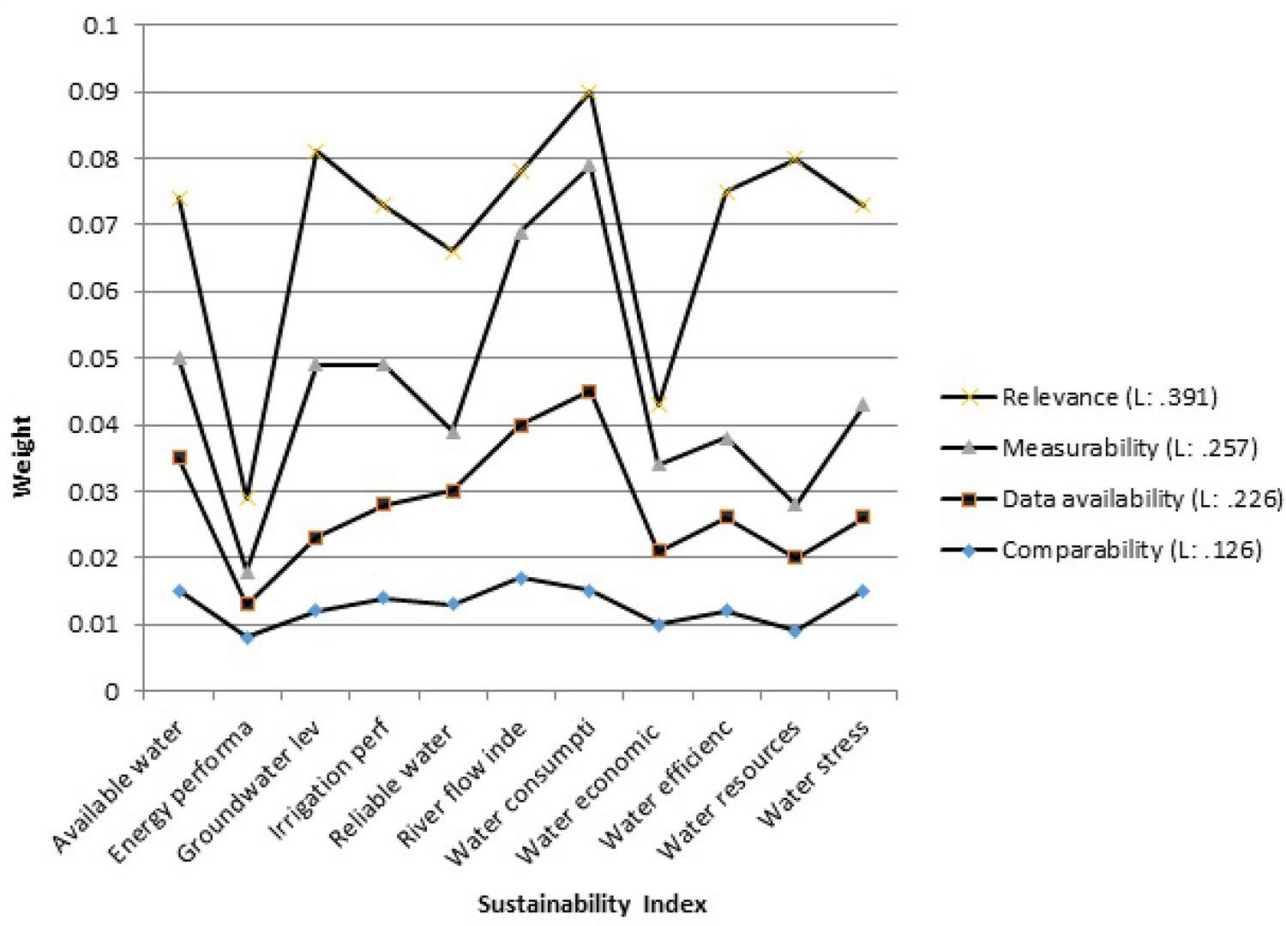
Water sustainability indicators.

This study compares the indicators water stress, river flow index in the dry season, reliable water supply, groundwater level sustainability, irrigation performance index, water consumption per kg of product, available water index, water efficiency index (kg m^−3^), water economic efficiency index, and water resources vulnerability index in the water sector. According to Fig. 5 the water consumption index per kg of product had the highest weight (0.09) in the sustainability of the agricultural system at the basin scale. This index shows how much water is input to produce one kilogram of agricultural product or livestock product, such as meat and dairy products. This SPI measures the water footprint of agricultural production in the study area. Fresán et al.^44^ used water consumption to evaluate the environmental impact of meat production^1^ concluded the main cause of food insecurity in the United States is the large amount of water-intensive crops that grow in areas with severe levels of water stress. Another dimension of water sustainability concern food waste, which means wasted water and energy. Agricultural sustainability is improved by the reduction of wasted food^45,46^.

Another indicator of sustainability is groundwater sustainability. This index can be evaluated with performance indicators^34^. There are indicators of vulnerability of water resources which evaluate the status of water resources sustainability. Excessive extraction and degradation of aquifers and the need of the agricultural sector to ensure food security will ultimately lead to reduced production and food insecurity. The river flow index in the dry season, for instance, was developed by^31^ to assess water conditions in river basins. This SPI calculates the temporal change in water access, for example during the season. Dry season basins are those that have less than 2% of their surface runoff in four of the driest months of the year. All SPIs that are related to the water sector are indicated in Fig. 5.

Improving farmers' water consumption management, in addition to increasing production and productivity of production inputs and improving farmers' incomes, increases farmers' access to a diverse and quality food basket and ultimately improves their food security.

### The energy sector

Energy is of special importance as an input in the agricultural sector. A study of energy use in the agricultural sector shows that over the years the use of petroleum products and electricity has increased. Increasing energy use raises the stress on natural resources. The importance of energy sustainability became apparent with the energy crises in the 1970s^8^. One of the 17 goals of the UN’ SDGs is to achieve affordable, reliable, sustainable, and modern energy.

**Figure 6:**
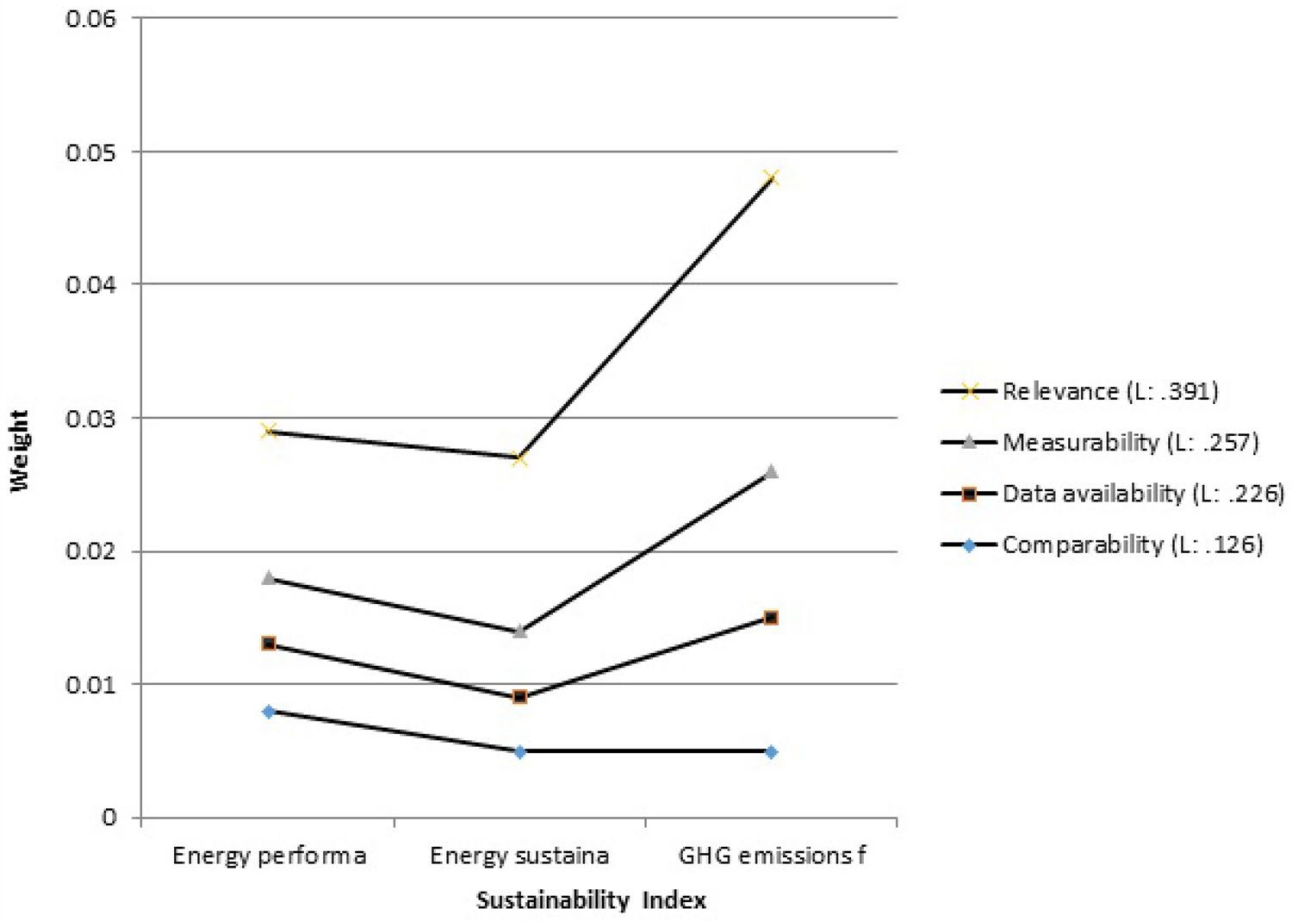
Energy sustainability indices.

Figure 6 compares the weights for the selected SPIs in the energy sector. It is seen in Fig. 6 the greenhouse gas (GHG) indicator had the first rank compared to the other two selected SPIs of the energy sector. This index was determined based on tier 1 and tier 2 methods of the Intergovernmental Panel on Climate Change—IPCC^26^. The lower the value of this index is, the more environmentally sustainable agriculture is^40^.

The second ranked energy sector SPI, i.e., the energy performance index, measures the intensity of energy consumption and is correlated with the factors influencing energy consumption. The third energy-related SPI is the energy sustainability index (ESI) that measures sustainable, cost-effective, and environmentally friendly energy supply and is widely used. In this this study the intensity of energy consumption has a larger weight than the ESI index.

### The food sector

**Figure 7:**
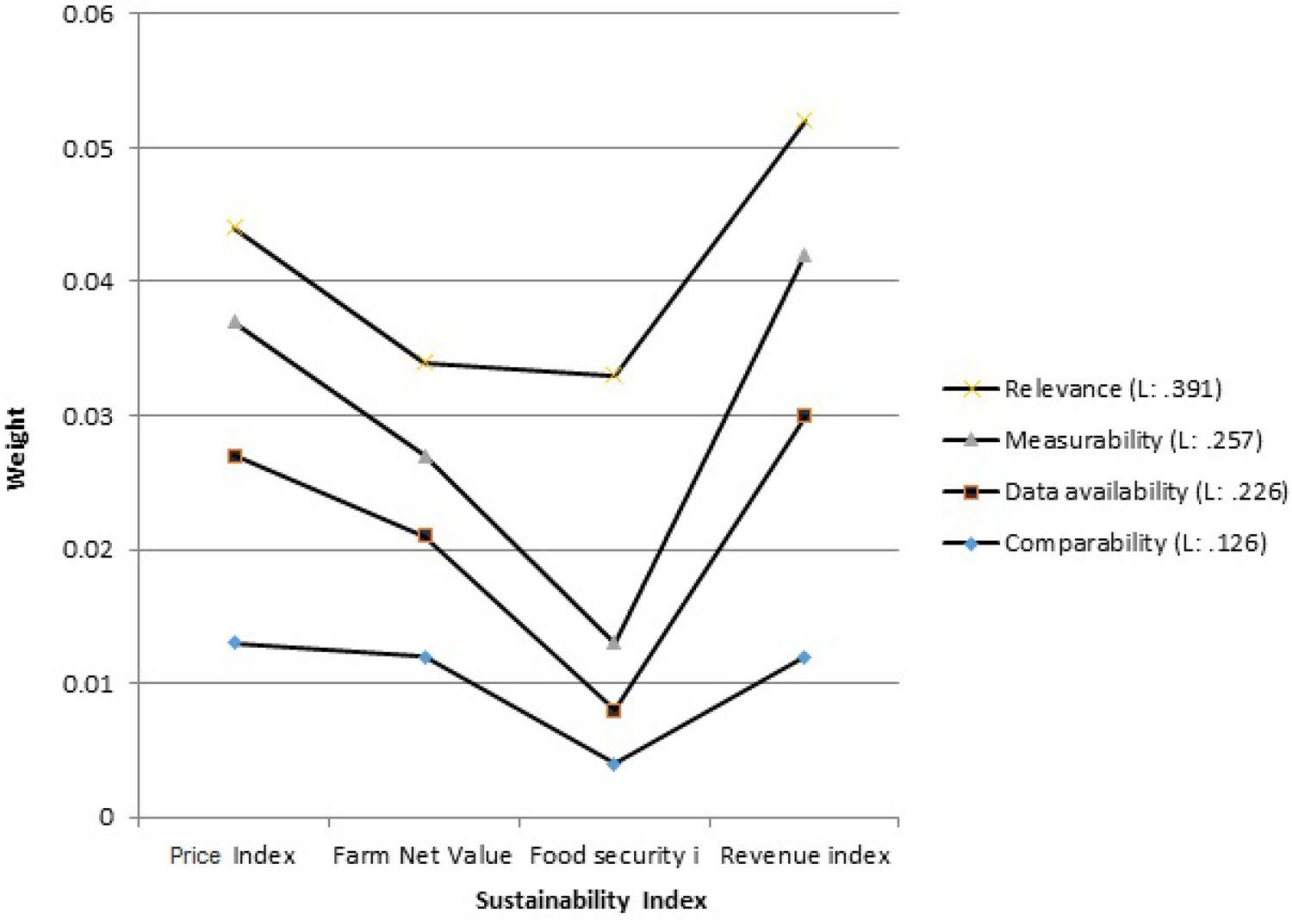
Food sustainability indices.

Revenue, price, farm net value, and food security indices were compared and ranked with other indicators. Among these four SPIs the revenue index and the price index had the highest and second highest weights, respectively. Figure 7 shows a comparison of the SPIs in the food sector. The high revenue rating in the agricultural sector is obvious, because when revenue exceeds cost farmers create wealth. Also, increasing farmers' incomes improves the management of agricultural water consumption and thereby increases farmers' food security. As a result, their access to food as well as their food security improves^47–49^).

The farm net value added (FNVA) index was ranked third in this ranking. This SPI measures the economic stability at the farm level. The FNVA index measures wealth creation by farming^40^. Evidently wealth creation must also lead to sustainability^50^.

The food security index was ranked fourth among these four SPIs. The Global Food Safety Index is often used by the FAO and other international organizations in the context of availability, accessibility, use, and stability^51^.

Food security, nutrition, and the eradication of poverty and hunger are also part of the UN’ SDGs. The activities related to the production and distribution of agricultural products generate employment and income. Yet, there are several challenges to economic sustainability in the agricultural sector, including high investment and operating costs or low incomes, while a stable system must be able to continuously supply the goods in demand. Revenue and price indicators can assess this dimension of the agricultural system as herein shown.

### Application of the selected SPIs

**Figure 8:**
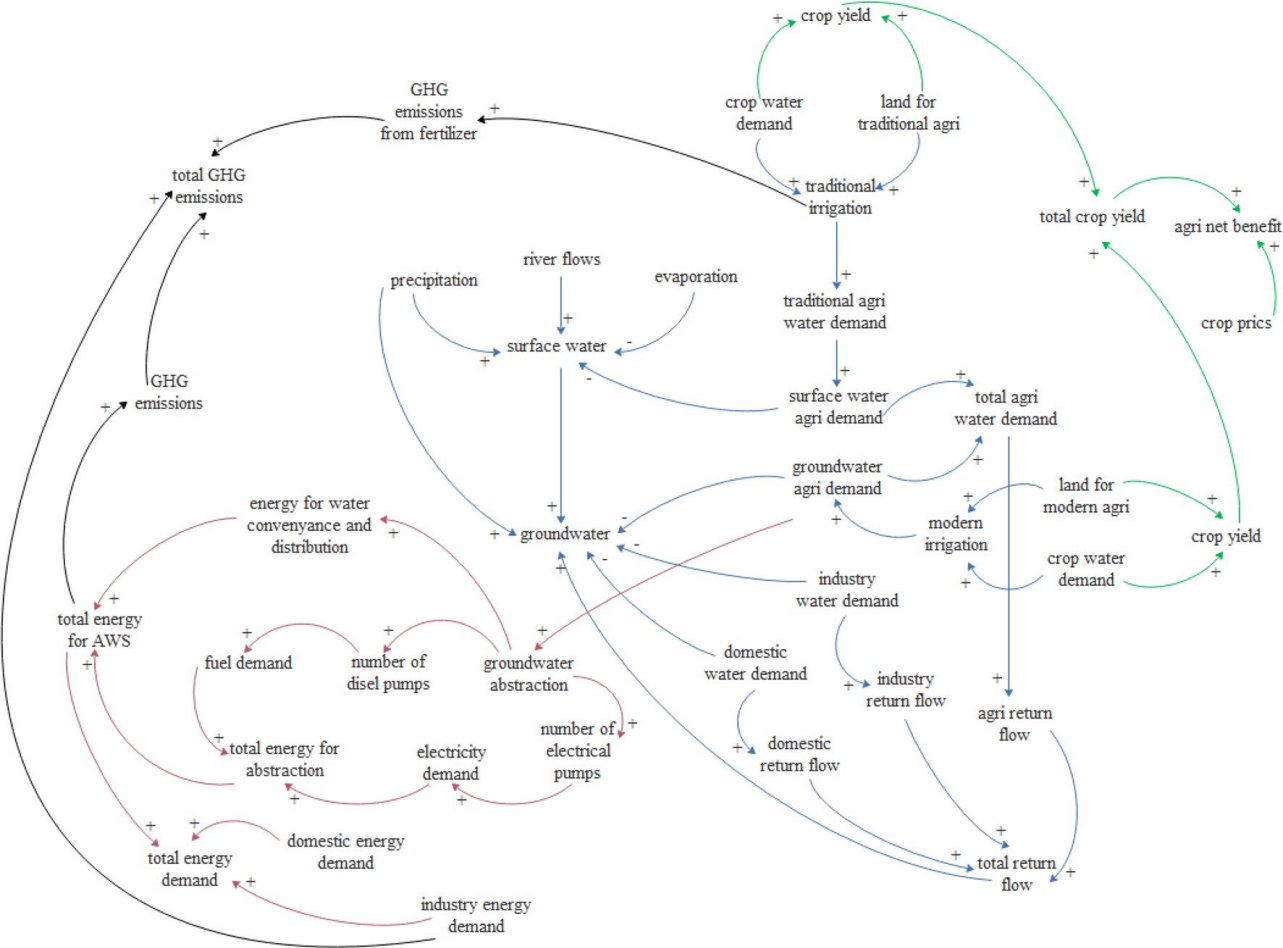
Interrelationships between the water-energy-food components of agricultural systems.

The Zayandeh-Rud basin was simulated with the system dynamics approach to investigate the application of the selected SPIs. SD considers the interactions between water, energy, and food parameters and variables (Fig. 8). SD was run for current situation and reducing 20% the under cultivation area and the best SPI, i.e. water consumption per kg of product, was calculated to compare the stability of the agricultural system under the scenarios. The results are presented in Table 5.

**Table 5:** The numerical value of the selected index in the Zaayandeh-Rud basin.

	Scenarios	Water consumption per kg of product (m^3^/kg)
Crops	1	0.11415
Horticultural products	2	0.0519
20% decrease in crops area	3	0.11372
20% decrease in horticultural products area	4	0.05216

Comparing the numerical value of this index for both crop and horticultural crops shows that the agricultural system is more sustainable with horticultural crops, and that by reducing the area under cultivation does not have much effect on the sustainability of the system. Therefore, modifying the cultivation pattern can be more effective in sustainability of agricultural systems than reducing the area under cultivation. This index considers the area under cultivation and the type of crop cultivated. Therefore, considering these key factors in an agricultural system reveals that this is an important indicator in the study of agricultural systems.

These scenarios were introduced to evaluate the superior index, and, clearly, more management scenarios could be defined. These results are useful and practical for determining the best management strategies to improve the sustainability of agricultural systems.

## Concluding remarks

Sustainable development meets the needs of the present generation without compromising the resources needed by future generations. Sustainable agricultural development avoids inefficient traditional and non-economic methods and relies on the use of modern agricultural knowledge and methods, and improves economic returns while achieving sustainability. Several SPIs corresponding to water, energy, and food sectors were selected. These SPIs were evaluated based on four criteria, i.e., relevance (importance), measurability, data availability, and comparability. The value of each SPI was determined based on expert’s judgment. The criteria were also ranked with the help of five scales (very high, high, medium, low, and very low). The AHP method was used to rank the SPIs based on 4 criteria and to select key indicators. The AHP is a powerful tool for solving decision making problems that are expressed as a hierarchy of levels. This work applied the AHP and found that the water consumption index, groundwater level sustainability index, water resources vulnerability index, and water stress index are effective indicators for assessing the stability of agricultural systems, along with revenue and carbon emission indicators. In addition to water resources SPIs in the energy and food sectors were also considered in this paper. The impacts of the energy and food SPIs were found to be smaller than that of water resources under the framework of this study. Other rankings might be obtained in other basins, yet, the comprehensive assessment of agricultural sustainability must consider the water-energy-food nexus.

## Data Availability

The data that support the findings of this study are available from the corresponding author upon reasonable request.
